# Impairments, health conditions and health risk behaviors: occurrence and associations, in the National Health Survey, Brazil, 2019

**DOI:** 10.1590/S2237-96222023000400002.en

**Published:** 2023-12-04

**Authors:** Marina Carvalho Arruda Barreto, Bárbara Bruna Rodrigues de Oliveira, Ileana Pitombeira Gomes, Mayra Solange Lopes de Vasconcelos, Nayranne Hivina Carvalho Tavares, Shamyr Sulyvan de Castro, Larissa Fortunato Araújo

**Affiliations:** 1 Universidade Federal do Ceará, Programa de Pós-Graduação em Saúde Pública, Fortaleza, CE, Brazil

**Keywords:** Disabled Persons, Chronic Disease, Health Risk Behaviors, Cross-Sectional Studies, Personas con Discapacidad, Enfermedad Crónica, Comportamientos de Riesgo para la Salud, Estudios Transversales, Pessoas com Deficiência, Doença Crônica, Comportamentos de Risco à Saúde, Estudos Transversais

## Abstract

**Objective:**

To analyze association of visual, hearing, mental/intellectual, physical and multiple impairments with health conditions and health risk behaviors in Brazil.

**Methods:**

This was a cross-sectional study, using data from the 2019 National Health Survey; associations between impairments and presence of cardiovascular disease (CVD), hypertension, diabetes *mellitus* (DM), high cholesterol, alcohol abuse and smoking were estimated using logistic regression, thus obtaining the odds ratios (OR).

**Results:**

Impairment was reported by 7.6% of the 90,846 participants. Having a impairment was associated with greater odds of reporting chronic conditions, especially CVD (OR = 2.11; 95%CI 1.76;2.54) and DM (OR = 1.78; 95%CI 1.56;2.02 ); visual impairment was associated with greater odds of smoking (OR = 1.52; 95%CI 1.28;1.81); mental/intellectual impairment was inversely related to smoking (OR = 0.45; 95%CI 0.30;0.67) and alcohol abuse (OR = 0.13; 95%CI 0.06;0.26).

**Conclusion:**

Having any of the impairments studied may be associated with greater odds of having chronic health conditions.

## Study contributions

**Main results:**

Positive association was identified between having one or more impairments, and health conditions and health risk behaviors. The higher the prevalence of mental/intellectual, physical and multiple impairments, the lower alcohol abuse and smoking.

**Implications for services:**

As this population is vulnerable to chronic health conditions, health services need strategies to reduce barriers to access, as well as health promotion actions, such as health education, adapted for people with impairments.

**Perspectives:**

Topics for future studies, examining the inverse relationship between impairments and alcohol abuse and smoking, in addition to acting on the causal chain, so as to prevent health conditions and health risk behaviors.

## RESUMEN

**Objetivo:**

Analizar la asociación entre discapacidad visual, auditiva, mental/intelectual, física y discapacidad múltiple con condiciones y conductas de riesgo para la salud en Brasil.

**Métodos:**

Estudio transversal utilizando datos de la Encuesta Nacional de Salud de 2019; se estimaron, mediante regresión logística, las asociaciones entre discapacidad con la presencia de enfermedades cardiovasculares (ECV), hipertensión, diabetes, colesterol alto, consumo de alcohol y tabaquismo, obtención del odds ratio (OR).

**Resultados:**

De los 90.846 participantes, el 7,6% refirió discapacidad. Tener una discapacidad se asoció con una mayor probabilidad de reportar enfermedades crónicas, especialmente ECV (OR = 2,11; IC_95_% 1,76;2,54) y diabetes (OR = 1,78; IC_95_% 1,56;2,02); la discapacidad visual se asoció con una mayor probabilidad de fumar (OR = 1,52; IC_95_% 1,28;1,81); la discapacidad mental/intelectual mostró una relación inversa con el tabaquismo (OR = 0,45; IC_95_% 0,30;0,67) y el consumo de alcohol (OR = 0,13; IC_95_% 0,06;0,26).

**Conclusión:**

Se observó que tener alguna de las discapacidades estudiadas puede estar asociado con mayores posibilidades de tener condiciones crónicas de salud.

## RESUMO

**Objetivo:**

Analisar a associação de deficiências visual, auditiva, mental/intelectual, física e deficiência múltipla com as condições e comportamentos de risco à saúde no Brasil.

**Métodos:**

Estudo transversal, utilizando-se dados da Pesquisa Nacional de Saúde de 2019; associações entre deficiências e presença de doença cardiovascular (DCV), hipertensão arterial, diabetes *mellitus* (DM), colesterol alto, consumo abusivo de álcool e tabagismo foram estimados por regressão logística, obtendo-se a razão de chances (RC).

**Resultados:**

Dos 90.846 participantes, 7,6% reportaram deficiência. Ter deficiência associou-se a maiores chances de relatar condições crônicas, especialmente DCV (RC = 2,11; IC_95_% 1,76;2,54) e DM (RC = 1,78; IC_95_% 1,56;2,02); deficiência visual associou-se a maior chance de tabagismo (RC = 1,52; IC_95_% 1,28;1,81); deficiência mental/intelectual apresentou relação inversa com tabagismo (RC = 0,45; IC_95_% 0,30;0,67) e consumo abusivo de álcool (RC = 0,13; IC_95_% 0,06;0,26).

**Conclusão:**

Apresentar alguma das deficiências estudadas pode-se associar a maiores chances de ter condições crônicas de saúde.

## INTRODUCTION

In 2019, approximately 1.5 billion people worldwide lived with some type of impairment, according to data relating mainly to low- and middle-income countries such as Brazil.^1^ Furthermore, generally speaking, levels of poverty and social exclusion are higher among people with impairments, which makes them even more vulnerable to health problems. Impairments may imply less access to health services, as these services often present physical accessibility and communication barriers associated with the stigma to be faced by this segment of the population.^2^

People with physical and mental impairments have greater difficulty in adopting health-promoting behaviors;^3,4^ for example, such impairments can lead to difficulties in purchasing food, impacting their food choices,^5^ in addition to difficulties in doing physical activities, due to environmental barriers and specific conditions of people with impairments.^4^ A study conducted in the United States, between 2002 and 2010, showed that people with physical and mental impairments were more prone to all types of substance abuse, including alcoholic beverages and tobacco.^6^ In this sense, having risk behaviors – i.e. use of these substances – could contribute to an increase in chronic health conditions.^2,3,7^

The United Nations Convention on the Rights of Persons with Impairments advocates people with impairments having the right to an adequate standard of living and social protection.^8^ Although it is more common for the impact of chronic diseases on the occurrence of impairments to be analyzed, the inverse relationship has been increasingly studied, based on social mechanisms that produce adverse health events in people with impairments.^9^

There are few studies that investigate association between the presence of impairment(s) and their multiple occurrence with unhealthy behaviors.^4^ However, the studies we did find were conducted in developed countries.^4,6^ One of those studies was conducted in the United States based on data from the national survey on drug use and health between 2002 and 2010, investigating the presence of impairments, without specifying the type of impairment, as well as the presence of multiple impairments, together with the occurrence of smoking, alcohol abuse and other drugs. Another of those studies was conducted in Australia using data from the 2015 National Health Survey. It investigated the relationship between physical, visual, hearing and mental impairments; but did not evaluate the simultaneous presence of these events with alcohol abuse, smoking, physical activity and obesity. The conclusion reached by both studies was that people with impairments are more prone to unhealthy behaviors.^4,6^

Understanding the association of different impairments with chronic health conditions and health risk behaviors offers a framework of essential information for health service management, including discussion of possible shortcomings in providing and ensuring access to health services and information. Studying possible association helps to understand which population profile most needs attention, based on the assumption of equity in health care and the permanent quest for better quality of life and longevity for the population. 

The objective of this study was to analyze association of visual, hearing, mental/intellectual, physical and multiple impairments with health conditions and health risk behaviors in Brazil. 

## METHODS

This was a cross-sectional study using data from the 2019 National Health Survey (*Pesquisa Nacional de Saúde* - PNS), conducted by the Brazilian Institute of Geography and Statistics (*Instituto Brasileiro de Geografia e Estatística* - IBGE), the Brazilian Ministry of Health and the Oswaldo Cruz Foundation (*Fiocruz*).^10^ The survey covered people aged 15 years or more.^10^

The National Health Survey uses a complex sampling plan, originating from a master sample, consisting of a plan comprised of clusters, with three selection stages, covering the entire national territory. The detailed description of the 2019 National Health Survey sampling definition methodology and the study design can be found in a specific publication.^11^ Data were collected from 94,114 households, with a 96.5% response rate. The inclusion criteria were: being aged 18 or over, availability of sociodemographic information and information on the occurrence of impairments, lifestyle, diseases and chronic health conditions. Participants under the age of 18 were excluded from the analysis; as were those who self-reported Indigenous race/skin color, as recommended by the IBGE, since they are small in number and their coefficient of variation is high. The final study population consisted of 90,846 participants. The freely accessible National Health Survey database can be found on the IBGE website [https://www.ibge.gov.br(accessed on December 18, 2022)].^10^

The study outcomes were cardiovascular disease (CVD), hypertension, diabetes *mellitus* (DM), high cholesterol, alcohol abuse and smoking. Information about these outcomes was obtained by directly asking the participants the following questions:

a) Has a doctor ever diagnosed you as having a heart disease, such as heart attack, angina, heart failure or other heart disease?;
b) Has a doctor ever diagnosed you as having hypertension (high blood pressure)? (not including pregnancy-induced hypertension);
c) Has a doctor ever diagnosed you as having diabetes? (not including pregnancy-induced diabetes);
d) Has a doctor ever diagnosed you as having high cholesterol? [module Q (Chronic diseases)]; and
e) In the last thirty days, have you consumed five or more doses of alcoholic beverages on a single occasion? (a dose of alcoholic beverage is equivalent to a can of beer, a glass of wine, a dose of white rum, whiskey or any other distilled alcoholic beverage) and Do you currently smoke any tobacco product? [module P (Lifestyles)].

The answer option for all these questions was “yes” or “no”.

The exposure variables were presence of visual, hearing, physical, mental/intellectual and multiple impairments – “yes” or “no” –, reported by means of the following questions:

a) Do you have permanent difficulty in hearing, even when using hearing aids? and *Do you have permanent difficulty in hearing?* (for hearing impairment);
b) Do you have permanent difficulty in seeing, even when using glasses, contact lenses or magnifying glasses? and *Do you have permanent difficulty in seeing?* (for visual impairment);
c) Do you have permanent difficulty in walking or going up steps, even using a prosthesis, walking stick or other walking aid?, Do you have permanent difficulty in walking or going up steps?, Do you have permanent difficulty in raising a bottle containing two liters of water from your waist up to eye level? and *Do you have permanent difficulty in picking up small objects, such as buttons and pencils, or opening and closing containers or bottles?* (for physical impairment, based on upper and/or lower limb impairment); and
d) Due to a mental or intellectual function limitation, do you have permanent in difficulty carrying out everyday activities, such as communicating, personal hygiene, working, going to school, playing, etc.? (for mental/intellectual impairment) [block G (People with impairment)].

The “multiple impairment” exposure variable was considered to exist when the person had two or more impairments (visual, hearing, physical; mental or intellectual).^12^

As they were related, both to exposures and to outcomes, the following variables were used for adjustments:

a) age (at last birthday: 18-24; 25-34; 35-44; 45-54; 55-64; 65 or over);
b)sex (male; female);
c) race/skin color (White; mixed race; Black; Asian);
d) schooling (completed higher education; completed high school education; completed elementary education; incomplete elementary education);
e) income [in minimum wages (MW): more than 5 MWs, 3-5 MWs, > 1-2 MWs, > ½-1 MW, > ¼- ½ MW; up to ¼ MW];
f) Brazilian macro-region (Southeast; South; Midwest; Northeast; North);
g) area of residence (rural; urban);
h) visit by community health worker (*agente comunitário de saúde* - ACS) or Family Health team in the last 12 months (never; once; 2-4 times; every 2 months; monthly); and
i) having health insurance (no; yes).

The characteristics of the population were presented using means or frequencies, with respective 95% confidence intervals (95%CI). The prevalence rates and 95%CI of health conditions and health risk behaviors were described according to the presence of visual, hearing, physical (upper and/or lower limbs), mental or intellectual impairment, and multiple impairments. The magnitudes of associations between types of impairments, as well as the occurrence of one or more impairments simultaneously, and health conditions and health risk behaviors were estimated by logistic regression, obtaining the odds ratios (OR) and their respective 95%CI as measures of effect, based on a 5% significance level. 

**Figure 1:**
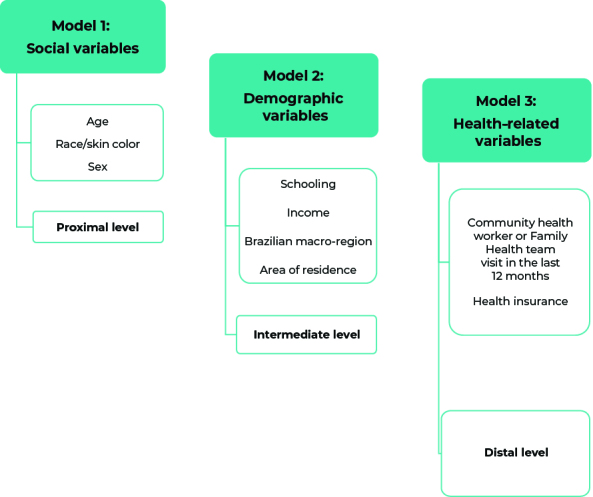
Hierarchical model of adjustments for associations between impairments and health conditions and health risk behaviors, National Health Survey, Brazil, 2019

Multivarible analysis of associations was performed using a hierarchical model, organized into three blocks of sequential adjustments, namely:

a) the first model included social aspect variables, such as age, race/skin color and sex – proximal level;
b) the second model was adjusted for demographic variables, such as schooling, income, Brazilian macro-region and area of residence – intermediate level; and
c) in the third model the two health-related variables were: a visit by a community health worker or Family Health team in the last 12 months; and having health insurance – distal level (Figure 1).

The analyses were adjusted according to these levels, taking a 5% significance level. 

The Hosmer & Lemeshow test was used to estimate the fit of the multivarible models. The statistical analyses were performed using Stata 15.0 (Stata Corporation, College Station, Estados Unidos), whereby complex sampling effects were considered using the survey command.

The 2019 National Health Survey project was submitted to the National Health Council National Research Ethics Committee, and was approved as per Opinion No. 3.529.376, dated August 23, 2019.

## RESULTS

Out of 94,114 people eligible for the study, 90,846 participants comprised the final sample. Losses corresponded to 3.5% that did not meet the inclusion criteria. Table 1 shows the characteristics of the population studied. There was a higher proportion of people in the 35-44 age group (20.5%), female sex (53.0%), mixed race (44.4%) and schooling level equivalent to incomplete elementary education (34.3%). Furthermore, 29.2% reported receiving between ½ and 1 minimum wage monthly; most of the interviewees (85.9%) lived in urban areas and 43.1% lived in the Southeast macro-region (Table 1).

**Table 1:** Sociodemographic, clinical and impairment characteristics in the study population (N = 90,846), National Health Survey, Brazil, 2019

Characteristics	% (95%CI^a^)
**Age (at last birthday)**	
18-24	14.0 (13.5;14.5)
25-34	18.3 (17.8;18.8)
35-44	20.5 (20.0;20.9)
45-54	18.1 (17.6;18.5)
55-64	15.2 (14.8;15.6)
≥ 65	13.9 (13.3;14.2)
**Sex**	
Male	47.0 (46.4;47.6)
Female	53.0 (52.3;53.5)
**Race/skin color**	
White	43.1 (42.4;43.8)
Mixed race	44.4 (43.7;45.0)
Black	11.5 (11.0;11.8)
Asian	1.0 (0.8;1.1)
**Schooling (completed levels of education)**	
Completed higher education	15.0 (14.3;15.5)
Completed high school education	33.3 (32.6;33.8)
Completed elementary education	17.4 (16.9;17.9)
Incomplete elementary education	34.3 (33.6;34.9)
**Income [in minimum wages (MW)]**	
> 5 MWs	5.0 (4.6;5.4)
> 3-5 MWs	6.2 (5.8;6.5)
> 2-3 MWs	9.0 (8.5;9.2)
>1-2 MWs	27.5 (26.9;28.1)
> ½-1 MW	29.2 (28.6;29.8)
> ¼-½ MW	14.9 (14.4;15.3)
≤ ¼ MW	8.2 (7.9;8.5)
**Brazilian macro-region**	
Southeast	43.1 (42.3;43.9)
South	14.6 (14.1;15.0)
Midwest	7.6 (7.3;7.8)
Northeast	26.7 (26.1;27.2)
North	8.0 (7.7;8.2)
**Area of residence**	
Urban	85.9 (85.5;86.3)
Rural	14.1 (13.6;14.4)
**ACS^b^ visit in the last 12 months**	
Never	23.3 (22.3;24.1)
Once	11.9 (11.3;12.3)
2-4 times	16.4 (15.7;17.0)
Every 2 months	10.5 (9.9; 11.1)
Monthly	37.9 (36.9;38.9)
**Health insurance**	
No	73.5 (72.6;74.2)
**Health conditions and health risk behaviors**	
Cardiovascular disease	5.0 (4.8;5.3)
Hypertension	24.7 (24.2;25.2)
Diabetes *mellitus*	8.2 (7.9;8.5)
High cholesterol	15.3 (14.8;15.7)
Alcohol abuse	16.5 (16.1;17.0)
Smoking	12.1 (11.7;12.5)
**Impairments**	
None	90.3 (90.0;90.6)
One	7.6 (7.2;7.8)
Two or more	2.1 (1.9;2.2)
Hearing impairment	1.3 (1.1;1.4)
Visual impairment	4.0 (3.8;4.2)
Physical impairment	5.7 (5.5;6.0)
Mental/intellectual impairment	1.0 (0.8;1.1)

a) 95%CI: 95% Confidence interval; b) ACS: *Agente comunitário de saúde* (community health worker).

 Among the health conditions assessed, 24.7% (95%CI 24.2;25.2) of participants reported having hypertension, 8.2% (95%CI 7.9;8.5) DM, 15.3 % (95%CI 14.8;15.7) high cholesterol and 5.0% (95%CI 4.8;5.3) CVD. Regarding health risk behaviors, 12.1% (95%CI 11.7;12.5) were smokers and 16.5% (95%CI 16.1;17.0) reported abusive consumption of alcoholic beverages. Regarding access to health services, 37.9% (95%CI 36.9;38.9) stated that they received a visit from a community health worker or member of the Family Health team monthly and 73.5% (95%CI 72 .6; 74.2) did not have health insurance (Table 1). 

Regarding impairments, 1.3% (95%CI 1.1;1.4) reported having hearing impairment, 4.0% (95%CI 3.8;4.2) reported having visual impairment, 5.7 % (95%CI 5.5;6.0) physical impairment and 1.0% (95%CI 0.8;1.1) reported mental/intellectual impairment. Of all people with impairments studied, 7.6% (95%CI 7.2;7.8) reported only one type of impairment, while 2.1% (95%CI 1.9;2.2) reported two or more simultaneous impairments (Table 1). 

Table 2 describes health conditions and health risk behaviors among those who reported having impairment. In all types of impairment, hypertension, followed by high cholesterol, were the chronic health conditions with the highest prevalence, in particular with regard to physical impairment [hypertension = 58.4% (95%CI 56.2;60.6) and high cholesterol, 32.4% (95%CI 30.2;34.6)]. Alcohol abuse and smoking had lower prevalence, particularly among those with mental/intellectual impairment [alcohol abuse = 1.7% (95%CI 0.9;2.9) and smokers = 7.5% (95%CI 5.5;10.3)]. Higher prevalence of health conditions and lower risk behaviors was found among those who reported more than one impairment, compared to those with only one impairment. 

**Table 2:** Prevalence of health conditions and health risk behaviors according to visual impairment, hearing impairment, physical impairment (upper and/or lower limbs), mental/intellectual impairment and multiple impairment in the study population (N = 90,846), National Health Survey, Brazil, 2019

Impairments	Cardiovascular disease	Hypertension	Diabetes *mellitus*	High cholesterol	Alcohol abuse	Smoking
	% (95%CI^a^)	% (95%CI^a^)	% (95%CI^a^)	% (95%CI^a^)	% (95%CI^a^)	% (95%CI^a^)
**Visual**	14.6 (12.8;16.7)	46.0 (43.3;48.7)	21.7 (19.4;24.1)	26.2 (23.9;28.7)	9.7 (8.4;11.2)	17.4 (15.6;19.4)
**Hearing**	18.5 (14.7;23.0)	54.1 (49.4;58.8)	19.7 (16.0;24.0)	27.1 (22.9;31.6)	9.2 (7.1;12.0)	10.3 (8.1;12.9)
**Physical**	18.5 (16.8;20.2)	58.4 (56.2;60.6)	25.0 (23.1;27.1)	32.4 (30.2;34.6)	4.5 (3.8;5.2)	13.5 (12.1;15.1)
**Mental/intellectual**	17.1 (12.8;22.3)	37.4 (31.8;43.3)	16.0 (12.5;20.3)	24.7 (19.8;30.4)	1.7 (0.9;2.9)	7.5 (5.5;10.3)
**Multiple disabilities**						
One	13.7 (12.3;15.1)	48.6 (46.7;50.6)	20.0 (18.4;21.7)	26.9 (25.1;28.8)	8.5 (7.6;9.5)	15.3 (13.9;16.7)
Two or more	21.7 (18.9;24.9)	57.7 (53.7;61.6)	27.1 (23.7;30.7)	32.3 (28.8;36.1)	3.4 (2.5;4.5)	11.8 (9.85;14.21)

a) 95%CI: 95% confidence interval.

Table 3 shows the associations between types of impairments and health conditions and health risk behaviors. After sequential adjustments for sex, age, race/skin color, schooling, income, Brazilian macro-region, rural/urban area and access to health services, the odds of people with physical impairment having CVD, hypertension, DM and high cholesterol were 2.39 (95%CI 1.99; 2.87), 1.78 (95%CI 1.56;2.03), 1.95 (95%CI 1.66;2.30) and 1.60 (95%CI 1.39;1.84 ), respectively, when compared to those without impairments. Similar results were found for visual impairment and when there was one impairment, or two or more impairments. Presence of mental/intellectual impairment was associated with lower odds of alcohol abuse (OR = 0.13; 95%CI 0.06;0.26) and smoking (OR = 0.45; 95%CI 0.30;0.67). Lower odds of alcohol abuse were also found when there was one physical impairment (OR = 0.48; 95%CI 0.38;0.60) and when there were multiple impairments (OR = 0.44; 95%CI 0.30;0. 64). 

**Table 3:** Associations between visual impairment, hearing impairment, physical impairment (upper and/or lower limbs), mental/intellectual impairment and multiple impairment, and occurrence of health conditions and health risk behaviors in the study population (N = 90,846), National Health Survey, Brazil, 2019

Impairments	Cardiovascular disease	Hypertension	Diabetes *mellitus*	High cholesterol	Alcohol abuse	Smoking
	% (95%CI^a^)	% (95%CI^a^)	% (95%CI^a^)	% (95%CI^a^)	% (95%CI^a^)	% (95%CI^a^)
**Visual**						
Model 0^b^	**3.53 (2.99;4.16)**	**2.72 (2.43;3.05)**	**3.34 (2.89;3.86)**	**2.04 (1.79;2.32)**	**0.53 (0.45;0.62)**	**1.56 (1.36;1.78)**
Model 1^c^	**2.16 (1.83;2.55)**	**1.39 (1.24;1.56)**	**1.91 (1.63;2.24)**	**1.37 (1.20;1.56)**	0.87 (0.72;1.03)	**1.66 (1.45;1.91)**
Model 2^d^	**2.18 (1.84;2.58)**	**1.32 (1.17;1.49)**	**1.81 (1.54;2.13)**	**1.40 (1.22;1.60)**	0.93 (0.78;1.13)	**1.45 (1.25;1.67)**
Model 3^e^	**2.01 (1.65;2.44)**	**1.26 (1.10;1.45)**	**1.71 (1.42;2.06)**	**1.35 (1.17;1.56)**	1.06 (0.84;1.34)	**1.52 (1.28;1.81)**
**Hearing**						
Model 0^b^	**4.42 (3.33;5.87)**	**3.66 (3.01;4.45)**	**2.79 (2.15;3.62)**	**2.08 (1.66;2.60)**	**0.51 (0.38;0.68)**	0.82 (0.64;1.07)
Model 1^c^	**1.97 (1.49;2.61)**	**1.47 (1.22;1.77)**	1.22 (0.93;1.60)	1.21 (0.96;1.53)	0.85 (0.61;1.17)	0.89 (0.68;1.16)
Model 2^d^	**1.94 (1.46;2.57)**	**1.42 (1.18;1.70)**	1.17 (0.89;1.54)	1.21 (0.95;1.53)	0.89 (0.64;1.15)	0.82 (0.63;1.07)
Model 3^e^	**1.53 (1.11;2.12)**	**1.37 (1.11;1.70)**	1.14 (0.85;1.53)	1.26 (0.95;1.67)	1.14 (0.79;1.65)	0.94 (0.69;1.29)
**Physical (upper and/or lower limbs)**						
Model 0^b^	**5.14 (4.52;5.85)**	**4.81 (4.38;5.27)**	**4.34 (3.85;4.90)**	**2.89 (2.58;3.24)**	**0.22 (0.19;0.26)**	**1.14 (1.00;1.30)**
Model 1^c^	**2.54 (2.21;2.92)**	**1.86 (1.68;2.07)**	**2.00 (1.76;2.28)**	**1.61 (1.43;1.82)**	**0.44 (0.37;0.53)**	**1.25 (1.08;1.44)**
Model 2^d^	**2.51 (2.18;2.90)**	**1.76 (1.59;1.95)**	**1.88 (1.65;2.15)**	**1.63 (1.44;1.83)**	**0.47 (0.39;0.57)**	1.05 (0.91;1.22)
Model 3^e^	**2.39 (1.99;2.87)**	**1.78 (1.56;2.03)**	**1.95 (1.66;2.30)**	**1.60 (1.39;1.84)**	**0.48 (0.38;0.60)**	1.11 (0.92;1.34)
**Mental/intellectual**						
Model 0^b^	**3.97 (2.83;5.58)**	**1.83 (1.43;2.34)**	**2.15 (1.60;2.89)**	**1.83 (1.38;2.43)**	**0.08 (0.04;0.15)**	**0.59 (0.41;0.83)**
Model 1^c^	**2.46 (1.68;3.59)**	1.00 (0.75;1.32)	1.26 (0.93;1.69)	**1.42 (1.08;1.87)**	**0.10 (0.05;0.19)**	**0.68 (0.48;0.96)**
Model 2^d^	**2.42 (1.65;3.51)**	0.93 (0.70;1.23)	1.15 (0.86;1.55)	**1.39 (1.05;1.83)**	**0.11 (0.06;0.20)**	**0.52 (0.36;0.74)**
Model 3^e^	**2.48 (1.49;4.12)**	0.74 (0.51;1.07)	1.22 (0.84;1.75)	1.24 (0.91;1.70)	**0.13 (0.06;0.26)**	**0.45 (0.30;0.67)**
**Multiple impairment**						
Model 0^b^						
One	**3.87 (3.38;4.44)**	**3.37 (3.10;3.66)**	**3.46 (3.07;3.89)**	**2.28 (2.05;2.54)**	**0.43 (0.38;0.49)**	**1.34 (1.19;1.50)**
Two or more	**6.78 (5.60;8.20)**	**4.86 (4.12;5.73)**	**5.12 (4.25;6.18)**	**2.97 (2.50;3.52)**	**0.16 (0.12;0.22)**	0.99 (0.80;1.22)
Model 1^c^						
One	**2.28 (1.96;2.64)**	**1.64 (1.50;1.80)**	**1.86 (1.63;2.12)**	**1.44 (1.28;1.60)**	**0.69 (0.60;0.79)**	**1.39 (1.24;1.57)**
Two or more	**3.19 (2.63;3.88)**	**1.70 (1.45;1.99)**	**2.24 (1.83;2.72)**	**1.68 (1.42;2.00)**	**0.32 (0.23;0.45)**	1.19 (0.96;1.49)
Model 2^d^						
One	**2.30 (1.98;2.66)**	**1.57 (1.43;1.72)**	**1.78 (1.56;2.02)**	**1.46 (1.31;1.63)**	**0.73 (0.63;0.84)**	**1.21 (1.07;1.37)**
Two or more	**3.23 (2.65;3.95)**	**1.58 (1.35;1.86)**	**2.07 (1.70;2.53)**	**1.71 (1.43;2.03)**	**0.35 (0.25;0.49)**	0.94 (0.75;1.19)
Model 3^e^						
One	**2.11 (1.76;2.54)**	**1.54 (1.38;1.72)**	**1.77 (1.51;2.08)**	**1.42 (1.24;1.61)**	**0.79 (0.66;0.95)**	**1.25 (1.07;1.45)**
Two or more	**2.89 (2.25;3.72)**	**1.50 (1.23;1.82)**	**2.06 (1.64;2.60)**	**1.65 (1.35;2.01)**	**0.44 (0.30;0.64)**	1.06 (0.81;1.40)

a) 95%CI: 95% confidence interval; b) Model 0 = without adjustment (crude model); c) Model 1 = Model 0 + adjustment for age, race/skin color and sex; d) Model 2 = Model 1 + adjustment for schooling, income, Brazilian macro-region and area of residence; e) Model 3 = Model 2 + visit by community health worker (*agente comunitário de saúde* - ACS) or Family Health team in the last 12 months + health insurance. Note: Values in bold type correspond to odds ratios (ORs) with p-value < 0.05.

## DISCUSSION

In this study based on national data, it could be seen that having some type of impairment, such as visual, hearing, physical, mental/intellectual impairment, may be associated with greater odds of having CVD, hypertension, DM and high cholesterol. Some magnitudes of association were stronger when two or more impairments were present simultaneously. An inverse association was found between presence of physical, mental/intellectual and multiple impairments, and smoking. Furthermore, presence of the impairments mentioned above was associated with lower odds of alcohol abuse. 

The results suggest that people who have some type of impairment may be more susceptible to developing chronic health conditions, compared to those without impairments. These findings reinforce what is presented in the National Health Policy for People with Impairments (*Política Nacional de Saúde da Pessoa com Deficiência*),^13^ namely that impairments can be risk factors for the development of health conditions, and thus point to the need for impairments to be the object of specific policies. 

The differences found in this study can be explained, in part, by socioeconomic aspects related to impairments, since they have already been associated with lower levels of schooling, lower employment rates, lower income and consequently, more precarious access to health systems and rehabilitation services, these being important factors related to the management of good health conditions.^14^ Furthermore, socioeconomic factors have been identified as being relevant in association of physical/sensory impairments with health conditions, also leading to an increase in social exclusion and stress.^2,15^

Poorer living conditions have also been identified as being associated with the development of depression, anxiety and other mental disorders.^16^ This situation can result in behaviors that are harmful to health, such as abusive use of drugs (tobacco, alcohol) and excessive consumption of foods with high energy density, these being factors that are also related to the development of chronic health conditions.^16^

An United States study, carried out with 465 participants from the Psychology Department of the Louisiana State University in 2019, identified that anxiety is more common in people with visual impairment than in the general population.^17^ Taking this into consideration, anxiety could be a mediator between this impairment and smoking, as it has been related to the use of electronic cigarettes and tobacco in the general population.^18^

Some physiological conditions underlying impairments themselves could also play an important role in the relationship between impairments and greater odds of having cardiovascular disease, hypertension, diabetes *mellitus* and high cholesterol. Functional limitations and limitations with regard to activity and participation in this population, added to environmental barriers, can cause a sedentary lifestyle and consequently, a reduction in basal metabolic rate, positive energy balance and changes in body composition, resulting in progressive loss of muscle mass and an increase in adipose tissue, in addition to greater risk of developing chronic health conditions.^2,19-21^

When analyzing association between impairments and alcohol abuse, it can be seen that physical impairment and mental/intellectual impairment contribute to reducing the odds of adopting this behavior. Population studies carried out in the United States in 2011, and in Australia in 2015, showed that people with impairment were less prone to alcohol abuse.^4,6^ This finding can be related to limitations caused by impairment, such as reduced mobility, in some cases, greater dependence on third parties, and lower income,^15,22^ these being situations that result in less possibility of purchasing alcoholic beverages. Furthermore, a study carried out with data from the 2002 Multicentric Health Survey in the State of São Paulo (*Inquérito Multicêntrico de Saúde no Estado de São Paulo* - ISA-SP), and data from the 2003 Health Survey in the Municipality of São Paulo (*Inquérito de Saúde no Município de São Paulo* - ISA-Capital)^23^ identified that people with physical impairments consumed 20% more medications than people without impairments; and that diuretics and analgesics were among the most used medications,^24^ with which alcohol could interact. As this is a possible factor in reducing alcohol consumption, new research is needed in order to deepen knowledge on the topic.

Environmental barriers to access to health services are also something to question, as a contributory factor to the greater vulnerability of the health conditions of people with impairments, since impairment can limit access and thus hinder seeking care.^14,22,25^ A study carried out in Chile, with data from the 2013 National Health Survey, demonstrated that people with physical impairment were three times more likely to report mobility difficulties in getting care at health services.^25^ In Brazil, a survey carried out in 38,811 primary health centers in 5,543 municipalities, between 2012 and 2013, showed that only 21% of services had professionals trained to care for service users with sensory impairments, and only 1% of health centers had auditory resources and support material available.^15^ As such, people with impairments could encounter difficulties in accessing health information, which would also contribute to greater vulnerability in relation developing chronic health conditions and adopting health risk behaviors.

This study has some limitations. Standing out among them is reverse causality, which does not allow a causal relationship to be established between exposures and outcomes. Therefore, it is possible that the health conditions investigated appeared temporally, before the occurrence of impairments. In the 2019 edition of the National Health Survey no data were collected regarding the nature of impairments (congenital or acquired), which could clarify the temporal relationship between exposures and outcomes assessed. Furthermore, as the information on impairments gathered by the National Health Survey was self-reported, i.e. provided by the participants themselves, this could lead to distortions such as underestimation or overestimation, due to inadequate understanding of the questions, especially in the case of participants with more pronounced mental or intellectual impairments.

Despite the aforementioned limitations, this study has strengths to be considered, with emphasis on investigation of multiple impairments and their simultaneous relationship, shedding light on the possible cumulative impacts of these impairments on the outcomes found. Furthermore, several outcomes were analyzed, which could help to gain a better understanding of the relationship between impairments, chronic health conditions and risk behaviors. It is worth highlighting that, even after adjusting for possible confounding elements, the associations found remained, which indicates the robustness of the findings.

In conclusion, impairments were associated with greater risk of chronic health conditions, a possible result of shortcomings in health information accessibility and access to services. Positive and negative associations were found with regard to adopting health risk behaviors, indicating that some impairments can protect against alcohol abuse and smoking. Thus, in order to achieve greater equity in comprehensive health care for people with impairments, it is necessary to take a more discerning look at the control and prevention of chronic health conditions and health risk behaviors, with the aim of ensuring healthy aging, with greater functioning for those with impairment.
